# 

**Published:** 1962-04

**Authors:** 


### Contents

IATROGENIC LIVER DISEASE . . . . . *A. E. Read* 41

CONGENITAL MALFORMATIONS . . *Beryl D. Corner* 46

CONGENITAL MALFORMATIONS . . . *N. J. Brown* 53

UNUSUAL TYPE OF BACTERIAL ENDOCARDITIS IN A CASE OF MYOCARDIAL INFARCTION . . *Clinico-Pathological Conference* 60

NOTES AND NEWS . . . . . . . . . 65

EXAMINATION RESULTS . . . . . . . . 65

APPOINTMENTS . . . . . . . . . 65

NEWS FROM THE SOCIETIES . . . . . . . 67

